# Anesthetic management for orthognathic surgery under ERAS protocol: a narrative review

**DOI:** 10.3389/froh.2026.1738483

**Published:** 2026-05-29

**Authors:** Ruikun Wang, Yu Liu, Wenwen Zhang, Jieqiang Zhang, Luping Wang

**Affiliations:** 1 Department of Anesthesiology, Hospital of Stomatology, Jilin University, Changchun, China; 2 Department of Anesthesiology, The First Hospital of Jilin University, Changchun, China

**Keywords:** anesthesia, Enhanced RECOVERY AFTER SURGERY (ERAS), intraoperative hemorrhage control, multimodal analgesia, orthognathic surgery, postoperative nausea and vomiting (PONV) prevention

## Abstract

**Objectives:**

This review aims to analyze the evolution and implementation of Enhanced Recovery After Surgery (ERAS) protocols in orthognathic surgery, particularly focusing on anesthesia management including core components: multimodal analgesia, intraoperative hemorrhage control, postoperative nausea and vomiting (PONV) prevention, airway management, glucocorticoid application, and perioperative fluid management. The goal is to identify key strategies for improving patient recovery through evidence-based practices.

**Materials and methods:**

A narrative review of existing literature was conducted, analyzing studies published on ERAS and anesthesia management in orthognathic surgery from 1997 to the present.

**Results:**

This review highlights the progress of ERAS protocols, which optimize perioperative care by focusing on multimodal anesthesia management, controlled hypotension, and strategies to alleviate pain, nausea, and inflammation via the aforementioned anesthesia-specific interventions. It is evident that ERAS can reduce opioid consumption, shorten hospital stays, and improve overall recovery. However, no standardized ERAS protocol has been universally adopted for orthognathic surgery.

**Conclusions:**

ERAS protocols have shown promise in improving patient outcomes in orthognathic surgery, but further research and the development of a unified protocol with standardized anesthesia management specifications are needed. Multidisciplinary collaboration remains crucial to effectively implement ERAS practices.

**Clinical relevance:**

This review provides insights into the application of ERAS in orthognathic surgery and offers guidance for clinical practice, particularly regarding the optimization of anesthesia management with evidence-based anesthesia-specific ERAS interventions to promote faster recovery and minimize complications.

## 1 Introduction

Enhanced Recovery After Surgery (ERAS) refers to the adoption of a series of perioperative optimization measures supported by evidence-based medicine to reduce the physiological and psychological trauma stress of surgical patients, so as to achieve the goal of accelerating patient recovery. The rapid recovery of patients is the core concept of ERAS (1). In terms of anesthesia, it mainly includes patient education, perioperative multimodal analgesia and fluid management, as well as early postoperative rehabilitation training (2). ERAS has currently been applied in various departments and types of surgeries such as gastrointestinal surgery and gynecology, which can shorten the hospital stay of patients and improve their medical experience (3–7).

Orthognathic surgery is a surgical treatment method for dentofacial deformities. Epidemiological data indicate that approximately 5% of the general population present with severe dentofacial deformities requiring orthognathic surgical intervention, and the global annual volume of these procedures is steadily increasing (8). Through the combination of preoperative and postoperative orthodontic treatments, malpositioned teeth are corrected, disharmonious dental occlusion is adjusted, and an occlusion acceptable for postoperative orthodontics is established (8, 9). Orthognathic surgery is mainly applicable to patients with congenital developmental deformities (mandibular retrognathia, maxillary protrusion, mandibular prognathism), patients with deformities caused by trauma or diseases, and those who undergo surgery seeking improvement in facial aesthetics. It can improve facial contours, enhance facial aesthetics, and boost self-confidence and quality of life (9–11).

Due to the rich blood vessels and nerves in the operation range of orthognathic surgery, its bleeding sources include bone wound bleeding, bone marrow cavity bleeding, and vascular bleeding in soft tissues, etc. The intraoperative blood loss is considerable, and controlled hypotension is usually required to reduce intraoperative bleeding and ensure clear surgical field (12). Operations such as osteotomy can cause severe pain, requiring significant doses of analgesic drugs during the operation (13). The use of high-dose opioids may cause complications such as postoperative respiratory depression, nausea, and vomiting (14, 15). Therefore, comprehensive perioperative anesthesia management is crucial for the implementation and rehabilitation of orthognathic surgery. Although the clinical value of ERAS for orthognathic surgery has been preliminarily confirmed and its adoption rate in maxillofacial centers worldwide is growing rapidly, the current relevant evidence is still limited, and no consensus has been formed on key issues such as anesthesia management, drug selection, and optimization of analgesic regimens. Therefore, this review will focus on the core aspects of perioperative anesthesia management for orthognathic surgery and systematically summarize the practical progress and controversial focuses [including six aspects: anesthetic analgesia, hemorrhage control, prevention of postoperative nausea and vomiting (PONV), airway management, glucocorticoids, and perioperative fluid management] of the ERAS concept in anesthesia for orthognathic surgery.

## 2 Methodology

It should be explicitly stated that this article is a narrative review without a systematic selection process. However, to enhance transparency, our literature search strategy and criteria are detailed below. To identify literature relevant to anesthesia management within the context of orthognathic ERAS, we conducted a comprehensive search using PubMed, Embase, and the Cochrane Library. The search period spanned from January 1997, marking the initial proposal of the ERAS concept, to November 2025. We employed a combination of Medical Subject Headings and free-text terms, including “Orthognathic Surgery,” “Bimaxillary Osteotomy,” and “Enhanced Recovery After Surgery,” combined with specific anesthetic terms such as “Multimodal Analgesia,” “Controlled Hypotension,” “Postoperative Nausea and Vomiting,” and “Airway Management.”

Criteria for inclusion were peer-reviewed articles published in English, including randomized controlled trials (RCTs), prospective and retrospective cohort studies, and systematic reviews. We prioritized studies involving human participants undergoing Le Fort I osteotomy, bilateral sagittal split osteotomy (BSSO), or bimaxillary surgery. Specifically, we selected studies that evaluated anesthetic interventions relevant to the core components of ERAS, such as opioid-sparing analgesia, fluid optimization, and stress response reduction. We excluded case reports, editorials, animal studies, and papers where surgical technique rather than anesthetic management was the primary variable. Study selection was conducted by two independent authors, with discrepancies resolved through discussion to ensuring the narrative synthesis was based on robust evidence.

## 3 Implementation of the ERAS Protocol in Orthognathic Surgery

**Figure 1:**
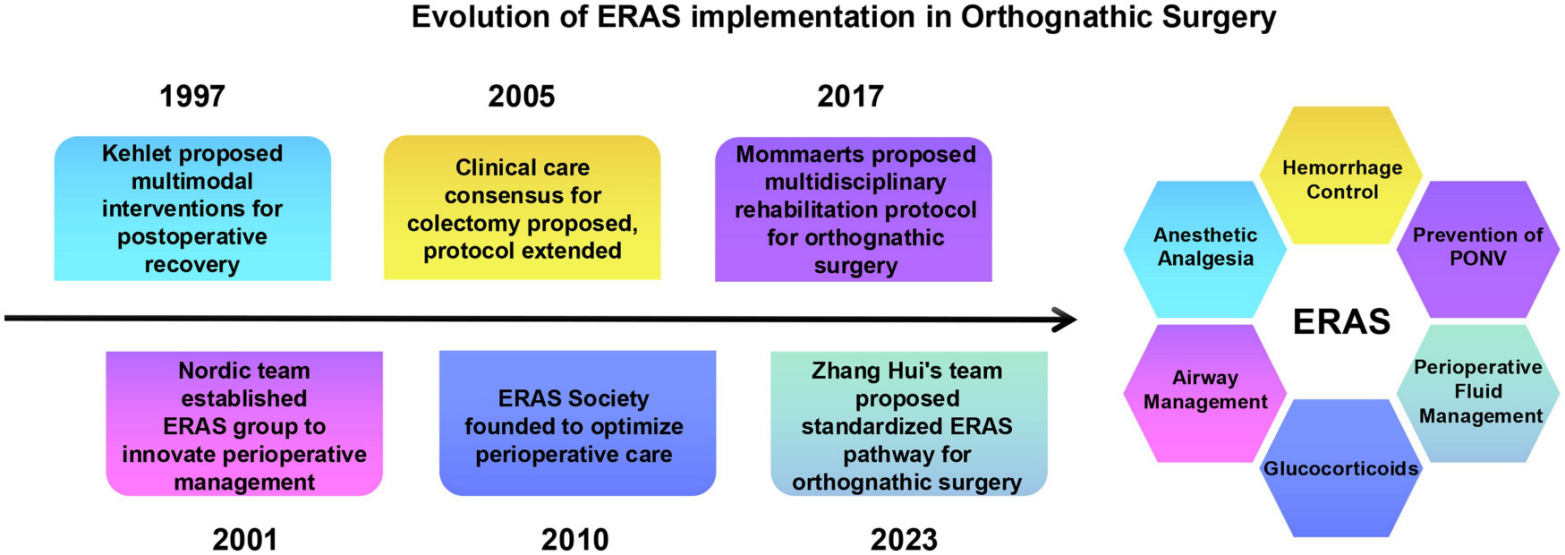
Evolution of ERAS implementation in orthognathic surgery.

The evolution of ERAS implementation in orthognathic surgery is shown in Figure 1. In 1997, after a thorough analysis of the influencing factors of perioperative complications and mortality in patients, Professor Kehlet put forward an innovative view: it is possible to promote the postoperative recovery process of patients and achieve the goal of early discharge by regulating the surgical stress response and adopting multimodal intervention measures (16). In 2001, six professors from five Nordic countries established an ERAS group to innovate the traditional perioperative management model based on evidence-based medicine. In 2005, the group first proposed a clinical care consensus for patients undergoing colectomy, optimizing perioperative management through a staged protocol to alleviate patient stress response, promote functional recovery, shorten hospital stay, and reduce healthcare costs, with the core goal of facilitating rapid postoperative recovery. Since then, the intervention protocol has been gradually extended to other surgical fields (17). In 2010, the ERAS Society was founded as a non-profit international multidisciplinary academic organization, aiming to optimize perioperative care and promote the implementation of global best practices through research and education (17, 18).

In 2017, Mommaerts et al. proposed a fast-track rehabilitation protocol involving multidisciplinary participation for the perioperative period of bimaxillary orthognathic surgery (19). The protocol constructed multiple measures involving preoperative, intraoperative and postoperative periods by reviewing the relevant problems of previous orthognathic surgeries. In recent years, a number of studies have also analyzed and summarized the aspects of preoperative education, perioperative pain management, controlled hypotension and prevention of PONV in the protocol. A retrospective study on patients who underwent bimaxillary orthognathic surgery found that the ERAS protocol could shorten the hospital stay of patients without increasing the readmission rate or the incidence of complications (20). However, there is currently no universally recognized ERAS protocol for orthognathic surgery. In 2023, Zhang et al. proposed a standardized clinical pathway for orthognathic surgery under ERAS (21). This protocol covers 27 perioperative intervention measures. A retrospective analysis of 658 patients undergoing orthognathic surgery found that ERAS interventions could shorten the postoperative hospital stay, reduce postoperative complications, and improve patient satisfaction. During the implementation of the above studies and protocols, anesthesia management, as a key component of perioperative care, has gradually highlighted its practical value in the ERAS concept. Therefore, this article summarizes the relevant contents of perioperative anesthesia management. Given that the original protocols encompass broad surgical techniques, nursing care, and dietary guidelines, we have extracted only the anesthesia-specific interventions from these recognized pathways (19, 21) to strictly align with the focus of this review. These extracted core anesthetic interventions, along with their clarified levels of evidence based on current literature, are summarized and refined in Table 1.

**Table 1:** Summary of anesthesia-specific ERAS interventions extracted from published protocols (19, 21) and their evidence levels.

Phase	Anesthetic Intervention	Main Objective	Level of Evidence
Preoperative	Preventive Analgesia (e.g., NSAIDs, Pregabalin)	Block central sensitization, opioid-sparing	High
	Preoperative Anxiolysis (e.g., Midazolam/Dexmedetomidine)	Reduce preoperative stress and anxiety	Moderate
Intraoperative	Total Intravenous Anesthesia (TIVA)	Reduce PONV incidence and severity	High
	Regional Nerve Blocks (Ultrasound-guided preferred)	Provide acute analgesia, reduce intra/post-op opioid use	Moderate
	Hemorrhage Control (Tranexamic acid + individualized moderate hypotension)	Reduce blood loss and transfusion need, preserve perfusion	High
	Airway Management (Lung-protective ventilation & awake extubation)	Prevent atelectasis, ensure airway safety during emergence	Moderate
	Fluid Management (Goal-directed or zero-balance)	Optimize tissue perfusion, prevent pharyngeal/facial edema	Moderate
	Glucocorticoids (Individualized administration based on risk)	Reduce postoperative edema, prevent PONV	Moderate
Postoperative	Multimodal Analgesia (NSAIDs, Acetaminophen, etc.)	Pain control, minimize opioid-related complications	High
	PONV Prophylaxis (Multimodal: e.g., 5-HT3 antagonists)	Prevent delayed PONV, facilitate early oral intake	High

## 4 Anesthesia management for orthognathic surgery

### 4.1 Anesthetic analgesia

#### 4.1.1 Multimodal analgesia

Perioperative pain management in orthognathic surgery is a key link in the implementation of ERAS. Multimodal analgesia reduces the dosage of a single drug, enhances the overall analgesic effect, and reduces adverse drug reactions by combining drugs with different mechanisms and different analgesic methods (22, 23). Specific measures include the application of non-steroidal anti-inflammatory drugs, opioids and other drugs, regional nerve block, patient-controlled analgesia, as well as physical analgesia such as ice pack cold compress and phototherapy, and psychological interventions (Table 2) (24). Among them, the commonly used analgesic drugs and their dosages are shown in Table 3 (23). Furthermore, dexmedetomidine, a highly selective alpha-2 agonist, serves as an excellent adjunct in multimodal regimens, significantly reducing opioid consumption while providing sedation without respiratory depression. When utilizing non-steroidal anti-inflammatory drugs (NSAIDs), clinicians must remain vigilant regarding their potential to impair platelet function and increase surgical bleeding risks. Therefore, for orthognathic patients at higher risk of bleeding, prioritizing selective COX-2 inhibitors may offer a safer alternative compared to non-selective NSAIDs (23). Given the significant concern of opioid abuse and the more obvious short-term postoperative demand for them in young women, multimodal analgesia can significantly reduce their dosage (25). In addition, vitamin C supplementation may help with postoperative pain management, as it may be consumed due to surgical stress and is associated with the dosage of postoperative analgesic drugs (26). In addition to commonly used drug analgesia and nerve block, the use of ice packs or ice towels after surgery as a physical analgesic method can reduce the patient's postoperative pain score and alleviate facial swelling; phototherapy intervention has also been proven to reduce postoperative pain and promote wound healing (27). In summary, perioperative pain management for orthognathic surgery takes multimodal analgesia as the core, combined with drugs, nerve block, physical intervention, and psychological support, which helps to reduce the patient's stress response, promote postoperative recovery, and optimize patient comfort and satisfaction (28).

**Table 2:** Common pain relief methods in orthognathic surgery .

Pain Relief Method	Specific Medications
Opioid Analgesics	Morphine, Fentanyl, Sufentanil, Remifentanil
Non-Steroidal Anti-Inflammatory Drugs (NSAIDs)	Non-selective COX inhibitors (e.g., Ibuprofen, Ketorolac)
	Selective COX-2 inhibitors (e.g., Celecoxib, Parecoxib)
Adjunct Analgesics	Calcium channel modulators (e.g., Gabapentin, Pregabalin)
	Corticosteroids
	NMDA receptor antagonists (e.g., Ketamine, Es-Ketamine)
Nerve Block Analgesia	Maxillary nerve block, Mandibular nerve block (Inferior alveolar nerve block)
Local Injection Analgesia	Infiltration anesthesia
Patient-Controlled Analgesia	Intravenous PCA (PCIA): Primarily opioid-based
Non-Pharmacological Analgesia	Physical therapy (e.g., Transcutaneous electrical nerve stimulation), Cryotherapy, Phototherapy
	Psychological intervention: Psychological counseling, Relaxation training, Cognitive-behavioral therapy

**Table 3:** Commonly used analgesic medications and dosages .

Drug	Route	Dosing (mg)	Interval (h)
Acetaminophen	Oral	325–625	Q 4–6
Celecoxib	Oral	Loading 400	Q 12
		Maintenance 200	
Ibuprofen	Oral	400–800	Q 6–8
Naprosyn	Oral	250–500	Q 12
Ketorolac	Intravenous	30–60	Q 6
Pregabalin	Oral	Initial 75	Q 12
		Max 150	
		Increase as needed	
Gabapentin	Oral	Initial 300	Q 24
		Max 600	Q 8
		Increase as needed	

#### 4.1.2 Preventive analgesia

To clarify terminology, preemptive analgesia specifically refers to interventions administered before incision to block central sensitization, whereas preventive analgesia is a broader concept encompassing any perioperative regimen aimed at minimizing sensitization, regardless of timing (29). Within ERAS protocols, preemptive analgesia is often regarded as a key component of a comprehensive preventive analgesic regimen. Specifically, preemptive analgesia emphasizes drug administration before surgery, whereas preventive analgesia encompasses interventions implemented at any time point throughout the perioperative period (30, 31).

Recent literature has validated these approaches. Regarding preemptive interventions, Lages et al., in a systematic review, reported that preemptive regimens involving dexamethasone or NSAIDs effectively controlled pain in periodontal surgery (32). Similarly, Ramasubbu et al. confirmed that a single preoperative dose of pregabalin—acting as a preemptive analgesic—significantly alleviated acute pain following orthognathic surgery (33). Canpolat et al. investigated the efficacy of intravenous ibuprofen for preventive analgesia in orthognathic surgery. Notably, their protocol involved administering the drug 30 minutes preoperatively, demonstrating that intervention implemented before the onset of noxious stimuli effectively reduced postoperative opioid consumption (34). This aligns with the findings of Alyahya et al., whose meta-analysis on various analgesic protocols highlighted that substantial reductions in postoperative pain scores are achievable when analgesic coverage is initiated prior to tissue injury (35). However, in comparison with preventive analgesia, some researchers argue that the effect of a single-dose intervention within the paradigm of preemptive analgesia only persists until the pharmacological action of the drug subsides, and thus fails to fully prevent central sensitization induced by perioperative pain. These researchers also emphasize that preventive analgesia implemented throughout the entire perioperative period can achieve a more favorable preventive effect (30). Therefore, the early initiation of multimodal analgesic interventions during the perioperative period is of critical significance for optimizing the postoperative recovery process in patients undergoing orthognathic surgery.

#### 4.1.3 Nerve block

Effective perioperative pain management not only directly affects patients’ postoperative recovery but may also reduce the risk and severity of chronic pain by alleviating acute pain. The ERAS protocol can effectively reduce patient pain and opioid consumption. Nerve block, by precisely injecting drugs around the target nerve, can effectively block pain conduction, achieve local analgesia, and significantly reduce opioid requirements. Depending on the type of local anesthetic selected and the adjuvant combination, the analgesic duration can be extended to more than 6 hours. Nerve blocks for orthognathic surgery are typically trigeminal nerve blocks, including maxillary nerve block and mandibular nerve block. However, the literature presents seemingly conflicting results regarding their efficacy, which necessitates a critical analysis of the study designs and techniques employed. Oono reported a retrospective study finding that the application of perioperative nerve block can alleviate postoperative pain, especially acute postoperative pain (36). In contrast, a retrospective cohort study by Wu et al. suggested that maxillary and inferior alveolar nerve blocks did not alleviate moderate to severe postoperative pain within the first 24 hours after bimaxillary surgery (37). This discrepancy likely stems from methodological heterogeneity, particularly the technique of administration. High-quality evidence from prospective trials appears to favor the use of advanced guidance technologies. The Esquerré team carried out a prospective randomized controlled study—providing a higher level of evidence than retrospective cohorts—to evaluate combined maxillary-mandibular nerve blocks under ultrasound guidance. Their results showed that this precise technique could reduce the postoperative oral morphine equivalent (OME) dosage by about 50% (38). This suggests that the inconsistent efficacy reported in earlier studies (like Wu et al.) may be attributed to the “blind” landmark-based technique, which has a higher failure rate compared to visualization-assisted blocks. Furthermore, the utility of nerve blocks extends beyond simple pain scoring. Even if pain scores are not drastically different, the opioid-sparing effect consistently observed across studies (including Wu et al.) is clinically significant because it directly reduces opioid-related complications such as postoperative vomiting (POV) (37). The study by Chen et al. found that effective trigeminal nerve block technology can not only reduce the dosage of analgesic drugs but also better facilitate the process of intraoperative controlled hypotension, decrease the intraoperative dosage of nicardipine, and simultaneously reduce intraoperative blood loss (39). In terms of practical implementation, current evidence suggests that the integration of nerve blocks into the ERAS protocol can be beneficial, with ultrasound guidance potentially improving success rates. However, recognizing the variability in operator experience and institutional practices, clinicians may view nerve blocks as a potential “bridging therapy” rather than a universal requirement. When utilized, they can provide high-quality analgesia during the immediate recovery phase, reduce intraoperative anesthetic depth, and help minimize opioid-induced nausea.

### 4.2 Hemorrhage control

Orthognathic surgery is typically associated with considerable blood loss, and perioperative bleeding is commonly managed through controlled hypotension, tranexamic acid administration, and other adjunctive measures (40). Controlled hypotension can reduce intraoperative bleeding and enhance surgical field visibility, but hypotension may decrease perfusion to vital organs and tissues, necessitating individualized adjustment based on patient status. A meta-analysis by Sakhariya's team demonstrated that controlled hypotension (maintaining mean arterial pressure at 50-65 mmHg) during general anesthesia is safe for young healthy patients undergoing orthognathic surgery. This approach reduces transfusion requirements by decreasing intraoperative blood loss and ultimately shortens hospital stays (12). Invasive blood pressure monitoring enables more precise blood pressure measurement and fluctuation control (41). The typical goals of controlled hypotension are: reducing systolic blood pressure to 80-90 mmHg, decreasing mean arterial pressure (MAP) to 70% of baseline, or maintaining it within the 50-65 mmHg range (42). However, strict patient selection is paramount. While this technique is proven safe for young, healthy individuals (ASA I-II), it should be strictly avoided or carefully modified in older patients and those with preexisting cardiovascular, cerebrovascular, or renal comorbidities (12). To prevent ischemic end-organ damage, the absolute safe limit of MAP should never fall below 50 mmHg. Furthermore, continuous invasive arterial blood pressure monitoring is strongly recommended during this process, as it provides real-time, precise hemodynamic data to ensure that these safe limits are strictly maintained (41). Tewari et al. compared factors associated with hypotensive anesthesia versus normotensive anesthesia during LeFort I osteotomy, concluding that controlled hypotension is a safe method for reducing intraoperative bleeding and improving surgical field visualization, though it showed no significant impact on operative duration or patient outcomes. The study also suggested that avoiding transfusion during the perioperative period may enhance hemorrhage control (43). This suggests that while hypotension facilitates the surgical technique, it must be carefully titrated to avoid masking tissue perfusion issues, which is contrary to the physiological preservation goals of ERAS. Particularly, deep controlled hypotension targets contradict the ERAS philosophy of maintaining physiological homeostasis, so blood pressure targets must be highly individualized. Commonly used pharmacological agents for controlled hypotension include inhaled anesthetics, sodium nitroprusside, nitroglycerin, remifentanil, calcium channel blockers, and *β*-blockers (44). Currently, the combination of remifentanil with propofol or clinically appropriate concentrations of inhaled agents (e.g., isoflurane, sevoflurane) has become the preferred widely adopted clinical protocol due to its safety profile, rapid onset, and short context-sensitive half-life, which aligns perfectly with the rapid recovery goals of ERAS (42, 43, 45–47). Crucially, the recent integration of tranexamic acid marks a shift in hemorrhage control strategies from “deep hypotension” to “physiological preservation.” In recent years, the application of tranexamic acid, a fibrinolysis inhibitor, has increased progressively in orthognathic surgery. Studies indicate that tranexamic acid reduces reliance on controlled hypotension during surgery, thereby mitigating the risk of hypoperfusion induced by hypotension, while effectively controlling intraoperative bleeding and shortening operative time (48). A recent meta-analysis conducted by Baghaie's team further corroborated these findings. The data revealed that compared to the control group, tranexamic acid administration resulted in an average reduction of 171.30 mL in intraoperative blood loss and 14.5 minutes in operative duration. The study confirmed that tranexamic acid not only significantly reduces intraoperative blood loss, shortens surgical time, and decreases transfusion risk, but also does not increase the incidence of thromboembolic complications (49). Finally, local vasoconstriction remains a vital adjunct in a multimodal hemorrhage control strategy. Singh's recent study demonstrated that administering tumescent solution 10 minutes before LeFortⅠ osteotomy reduces surgical bleeding, alleviates swelling pressure, and shortens postoperative hospitalization, providing novel insights for optimizing perioperative management (50). Based on this evidence, a practical ERAS approach should prioritize tranexamic acid and local measures to minimize bleeding, using only individualized and moderate controlled hypotension to protect organ perfusion.

### 4.3 Prevention of PONV

PONV is one of the most common adverse events following orthognathic surgery (51). Female sex, age <25 years, surgery duration >3 hours, history of motion sickness, and use of opioids or inhaled anesthetics are all recognized risk factors for PONV (52). The Apfel score is an effective tool for predicting PONV risk, featuring simple assessment with four risk factors (1 point each), where higher total scores indicate greater PONV probability (53). Based on this risk assessment, a multimodal prophylaxis algorithm may be considered: for example, utilizing single or dual antiemetic therapy for low-to-moderate risk, and escalating to triple therapy for high-risk patients. However, given that orthognathic patients typically possess multiple risk factors, a universal high-risk prophylactic approach is clinically preferable to selective screening. Severe PONV may lead to delayed wound healing, fluid/electrolyte imbalances, and prolonged hospitalization, with some patients reporting worse subjective distress than postoperative pain.

A retrospective cohort study by Pourtaheri's team confirmed high PONV incidence in orthognathic patients and suggested that antiemetics and reduced opioid analgesia may decrease postoperative nausea/vomiting (54). Most studies indicate that nerve blocks reduce perioperative opioid consumption, consequently significantly lowering PONV incidence (55). This confirms that opioid-sparing strategies are not merely for pain control but serve as the foundation of PONV prevention within the ERAS multimodal framework. 5-HT_3_ receptor antagonists are the most frequently used PONV prophylactics, with combined use with dexamethasone enhancing antiemetic efficacy (56). Compared to balanced inhalation anesthesia, total intravenous anesthesia reduces PONV and hemodynamic fluctuations during extubation/recovery (57). Therefore, despite the convenience of inhalational agents, Propofol-based TIVA may be considered a preferable option in ERAS protocols for this specific patient population, though the choice should ultimately be tailored to individual patient needs and clinical settings.

Recent studies reveal that certain sedatives and anticholinergic agents also suppress PONV. An El-Taher study on laparoscopic patients demonstrated midazolam's equivalent PONV prevention efficacy to ondansetron/dexamethasone (58). Perioperative dexmedetomidine is considered a viable option for reducing both postoperative pain and nausea in orthognathic surgery (59). Low-dose bolus plus continuous penehyclidine infusion effectively prevents PONV without increasing emergence agitation (60). ERAS protocols for orthognathic surgery should employ multimodal analgesia, antiemetics, and other approaches to reduce opioid use and PONV incidence while improving clinical outcomes (61–63). In practice, implementation may benefit from shifting towards a preventive strategy, often considering dual-antiemetic prophylaxis combined with TIVA, while acknowledging that treatment plans should remain flexible based on patient-specific risk profiles.

### 4.4 Airway management (protective lung ventilation)

The impact of intraoperative anesthesia management on postoperative outcomes has gained increasing attention, with individually titrated mechanical ventilation strategies playing a crucial role in enhanced recovery (64). Under general anesthesia, neuromuscular blocking agents reduce lung volume, alter ventilation/perfusion (V/Q) ratios, and contribute to atelectasis. Decreased functional residual capacity, airway closure, and high fraction of inspired oxygen are primary causes of atelectasis, while surgical trauma-induced inflammation increases ventilator-induced lung injury risk (65). In the specific context of orthognathic surgery, the routine use of narrower, longer nasotracheal tubes inherently increases airway resistance. Furthermore, surgical manipulation (such as maxillary impaction) can transiently compress the tube, leading to high peak inspiratory pressures, while blood and secretions in the surgical field heighten the risk of micro-atelectasis (65). Atelectasis significantly contributes to postoperative pulmonary complications, potentially prolonging hospitalization and increasing economic burdens. Lung-protective ventilation strategies prevent alveolar overdistension and atelectasis through low tidal volumes and optimal positive end-expiratory pressure (PEEP) (66). A meta-analysis indicates that intraoperative low tidal volume (6-8 mL/kg predicted body weight) ventilation with PEEP and intermittent recruitment maneuvers significantly improves pulmonary outcomes and reduces hospital stays (64). The Individualized Open Lung Approach (iOLA) combines recruitment maneuvers and personalized PEEP setting, typically achieved through incremental PEEP titration in pressure-controlled ventilation, followed by multimodal PEEP optimization to prevent alveolar collapse (67). Müller-Wirtz et al. demonstrated that inhaled anesthetics better preserve respiratory drive than intravenous agents, reducing lung stress/strain and ventilator-induced injury by maintaining protective inspiratory patterns, thus facilitating weaning in prolonged mechanical ventilation (68), a critical synthesis with the PONV literature suggests that for orthognathic ERAS, the priority of minimizing nausea often supersedes these respiratory benefits. Therefore, practically, lung-protective ventilation strategies should be rigorously applied under TIVA maintenance to ensure both lung protection and PONV prevention.

Furthermore, orthognathic surgery poses unique upper airway risks. Preoperatively, patients with craniofacial deformities (such as Crouzon syndrome) often present with difficult airway manipulation, necessitating meticulous pre-anesthetic evaluation and planned induction strategies (69). Postoperatively, skeletal movements in 2-jaw surgery directly alter oropharyngeal airway volumes and the cross-sectional area of the most constricted region (70). These anatomical changes, combined with postoperative pharyngeal edema and potential intermaxillary fixation (IMF), make emergency reintubation extremely difficult and hazardous. Therefore, ERAS pathways must emphasize strict awake extubation strategies (extubating only when the patient is fully awake with intact airway reflexes) alongside rigorous postoperative oximetry monitoring in a head-elevated position to manage potential airway emergencies.

### 4.5 Glucocorticoids

In general anesthesia, glucocorticoids play multifunctional roles, positively influencing patients’ intraoperative physiological status and postoperative recovery, typically including reducing inflammatory responses, preventing PONV, improving pulmonary function, suppressing excessive immune reactions, and providing adjunctive analgesia. Evidence suggests that incorporating glucocorticoids in multimodal analgesia protocols promotes postoperative recovery by reducing opioid requirements and adverse effects (71). A survey revealed that Finnish oral and maxillofacial surgeons routinely administer glucocorticoids in all orthognathic surgeries, believing they reduce postoperative swelling and pain (72). However, optimal dosing remains a subject of debate. Given the potential dose-dependent effect of dexamethasone on postoperative edema in orthognathic surgery, Abukawa et al. conducted a prospective RCT comparing 0 mg, 8 mg, and 16 mg dexamethasone, concluding that 16 mg was most effective for BSSO based on masseter thickness and maximum interincisal opening measurements (73). However, Lin et al. found no dose-dependent effects when analyzing different IV dexamethasone doses on facial swelling and PONV reduction in orthognathic surgery (74). This discrepancy likely stems from methodological heterogeneity, specifically the difference between linear measurements versus volumetric 3D photogrammetry. Bravo et al. demonstrated that perioperative IV glucocorticoids effectively reduce early postoperative edema (0-48 h) in orthognathic surgery without significant adverse effects (75). Additionally, glucocorticoids may decrease the incidence of moderate-to-severe early PONV. For orthognathic surgery, a 2-day postoperative dexamethasone regimen effectively reduces edema and enhances patient comfort (76). Furthermore, the PADDI trial (*n* = 8478), a multicenter RCT by Corcoran et al., established that 8 mg perioperative IV dexamethasone doesn't increase 30-day surgical site infection risk, confirming no increased risk of infectious complications (77). Clinicians should carefully weigh the anti-edema and anti-emetic benefits against patient-specific risks, ensuring that dosing strategies are discussed with caution and highly individualized, rather than universally adopting a high-dose protocol.

### 4.6 Perioperative fluid management

Perioperative fluid administration significantly impacts patients’ physiological function and postoperative recovery. Fluid therapy aims to optimize stroke volume and treat hypovolemia, replenish preoperative fluid deficits, maintain normal blood volume and acid-base balance during and after surgery, ensure adequate tissue/organ perfusion and oxygenation, thereby facilitating stable recovery and reducing complications (78). A systematic review by Shah's team demonstrated that excessive fluid therapy may lead to free flap-related complications or prolonged hospitalization in free flap patients, while a restrictive fluid strategy (intentionally limiting fluid intake to achieve a net negative or minimal positive balance) increases risks of delayed-onset thrombotic events (79). To clarify, zero-balance fluid therapy (replacing exactly what is lost to maintain preoperative body weight and normovolemia) is currently considered the optimal goal, with balanced crystalloids being the recommended perioperative fluid (80). In orthognathic surgery, strict adherence to zero-balance is particularly critical not just for homeostasis, but to minimize pharyngeal edema which directly threatens post-extubation airway patency. For high surgical-risk patients, goal-directed therapy (GDT)—which utilizes advanced hemodynamic monitoring (e.g., stroke volume variation) to precisely tailor fluid boluses and vasopressors to real-time individual physiological demand—is recommended. In practice, fluid and vasoactive drug administration follows standardized algorithms to correct hypotension and low cardiac output. Precise monitoring and adjustment based on hemodynamic parameters maintain stable circulation, ensure vital organ perfusion, and reduce perioperative adverse events (81). GDT is also recommended for orthognathic surgery perioperative fluid management, with growing attention to adolescent orthognathic ERAS protocols. However, GDT has not been widely adopted due to technical barriers or insufficient awareness (82). Consequently, in the absence of advanced GDT monitoring, a practical restrictive fluid strategy combined with vasopressors to maintain the “dry” field required for surgery must be approached with caution and discussed with proper contextualization. This approach requires strict individualized assessment and close hemodynamic monitoring to ensure that achieving surgical visibility does not come at the cost of systemic or occult tissue hypoperfusion. A summary comparing these ERAS-based strategies with conventional care is presented in Table 4.

**Table 4:** Comparison of conventional care versus ERAS-based anesthesia management.

Aspect	Conventional Care	ERAS-based Management
Analgesia	Opioid-based analgesia	Multimodal and preventive analgesia
Regional Block	Not routinely used	Consideration of nerve blocks
Anesthesia Type	Inhalation anesthesia	Preference for TIVA when feasible
Hemorrhage Control	Deep controlled hypotension	Tranexamic acid and local vasoconstriction
PONV Prevention	Treatment of symptoms	Prophylactic multimodal prevention
Airway	Standard ventilation	Lung-protective ventilation
Fluid Therapy	Liberal fluid administration	Zero-balance or goal-directed therapy
Glucocorticoids	Variable use	Individualized administration

### 4.7 Integrated ERAS anesthesia pathway

**Figure 2:**
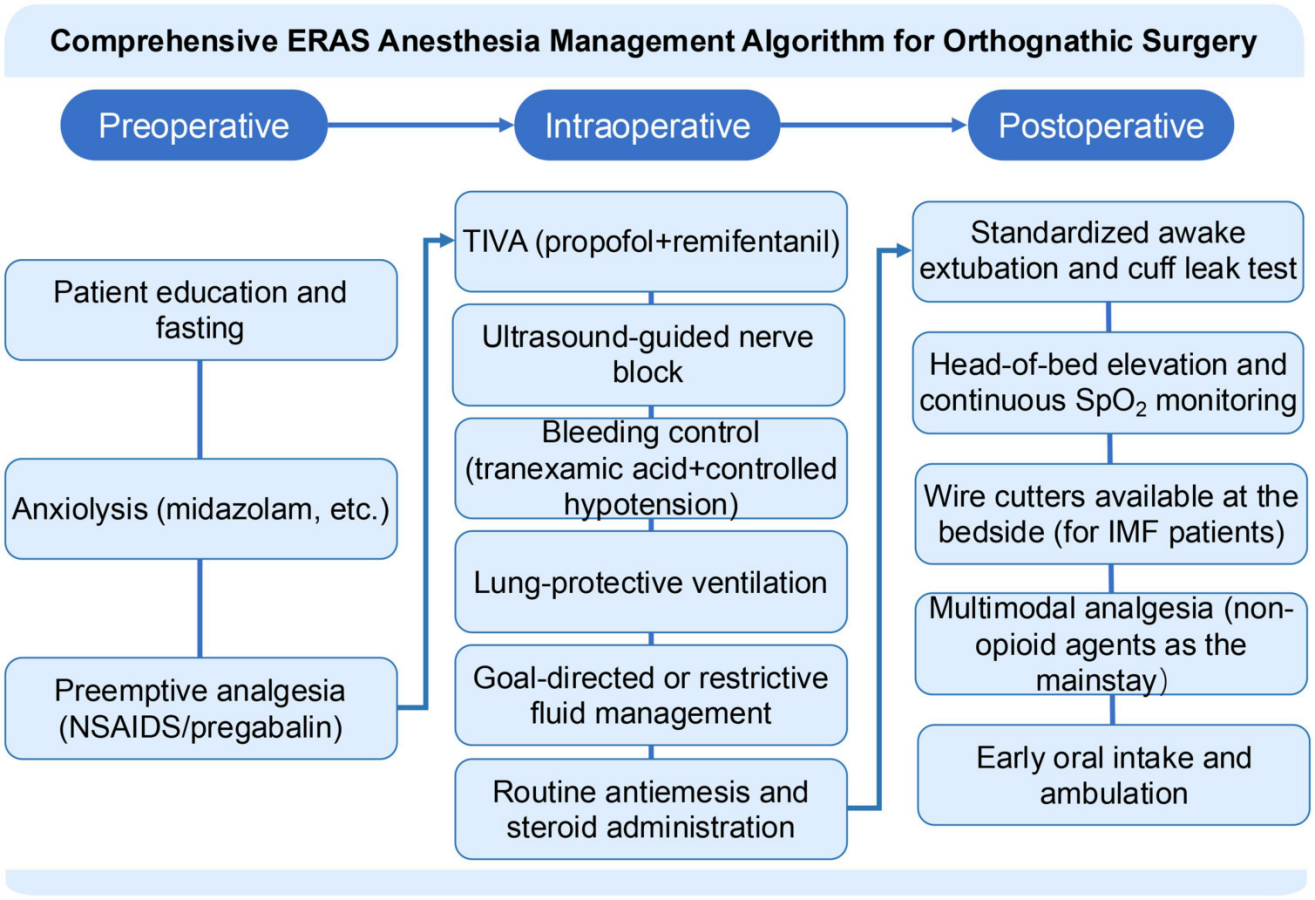
Comprehensive ERAS anesthesia management algorithm for orthognathic surgery. ERAS, enhanced recovery after surgery; TIVA, total intravenous anesthesia; NSAIDs, non-steroidal anti-inflammatory drugs; SpO_2_, peripheral oxygen saturation; IMF, intermaxillary fixation.

To synthesize the individual interventions discussed above into a clinically actionable framework, we have constructed a comprehensive perioperative anesthesia management algorithm for orthognathic surgery (Figure 2). This integrated pathway highlights the seamless transition from preoperative strategies (anxiolysis and preventive analgesia), through intraoperative management (TIVA-based maintenance, individualized blood pressure control, protective ventilation, and prophylactic antiemetics), to postoperative care (multimodal analgesia, strict awake extubation, and rigorous monitoring). This cohesive synthesis avoids the limitations of isolated interventions, aiming to maximally preserve physiological homeostasis, reduce complication risks, and accelerate postoperative recovery.

## 5 Limitations

Current evidence supporting anesthesia management in orthognathic ERAS is subject to several limitations. First, the literature is dominated by retrospective cohort studies with small sample sizes, which introduces potential selection bias and limits the strength of causal inferences compared to large-scale RCTs. Second, there is significant heterogeneity in ERAS protocols across institutions; variations in surgical techniques, analgesic regimens (e.g., specific nerve block approaches), and discharge criteria make direct comparisons difficult. Finally, a universally standardized ERAS pathway for orthognathic surgery remains absent, leading to inconsistent implementation of core elements such as fluid management and corticosteroid dosing. Future research should prioritize multi-center RCTs to establish consensus-based guidelines.

## 6 Conclusion

This review summarizes the implementation progress of anesthesia management under the ERAS concept in orthognathic surgery and highlights key aspects. Although the initial implementation with emerging evidence of ERAS in orthognathic surgery has shown potential in improving patient recovery, existing evidence remains limited, and consensus has yet to be reached on core issues such as anesthesia management, drug selection, and optimization of analgesic regimens. Anesthesia management measures, such as multimodal analgesia, controlled hypotension, glucocorticoid application, prevention of PONV, and airway management, play a crucial role in promoting postoperative recovery and reducing complications. Crucially, the success of these measures depends not on their isolated use, but on their synergistic application—such as pairing TIVA with preventive analgesia to simultaneously target pain and vomiting. As the ERAS concept continues to evolve, the formulation and optimization of anesthesia protocols provide a more personalized and safer treatment approach for patients. However, the standardization of ERAS implementation in orthognathic surgery is not yet complete, and the multidisciplinary collaboration model still needs further enhancement to ensure close coordination of various aspects and improve patient compliance. Future research should focus on refining the development of standardized ERAS protocols and promoting multidisciplinary collaboration to further improve the quality of surgery and anesthesia management, facilitating faster recovery for patients.

## References

[1] Nelson G Altman A Nick A Meyer L Ramirez P Achtari C. Guidelines for pre- and intra-operative care in gynecologic/oncology surgery: enhanced recovery after surgery (ERAS®) society recommendations — part I. Gynecol Oncol*.* (2016) 140:313–322. 10.1016/j.ygyno.2015.11.015 26603969

[2] Ljungqvist O de Boer HD Balfour A Fawcett WJ Lobo DN Nelson G. Opportunities and challenges for the next phase of enhanced recovery after surgery. JAMA Surg*.* (2021) 156:775–84. 10.1001/jamasurg.2021.0586 33881466

[3] Alivizatos V Gavala V Athanasopoulos P Apostolopoulos N. P-0154 enhanced recovery after surgery (eras) program versus conventional perioperative care in patients undergoing Major gastrointestinal surgical procedures. Ann Oncol*.* (2012) 23:iv75. 10.1016/s0923-7534(20)30076-4

[4] SSeguya P Arangelova S. A lady with a positive PET CT scan. Resp Med*.* (2025) 2:38–9. 10.52964/cfrm.0054

[5] Bergstrom J Alimi Y Scott M Tanner E Fader A Temkin S. 4: quality and safety in gynecologic oncology surgery as assessed through an enhanced recovery after surgery (ERAS) program. Am J Obstet Gynecol*.* (2017) 216:S562–S563. 10.1016/j.ajog.2016.12.155

[6] Abadir AF Benjamine FM Aziz AHF. A comparative study between traditional care program and enhanced recovery after surgery (ERAS) program in general surgery. QJM*.* (2020) 113:hcaa050.053. 10.1093/qjmed/hcaa050.053

[7] Plasencia E Mamani SC Schweer D Goicoechea JC. Decreased narcotic use after the implementation of ERAS guidelines in gynecologic oncology at an academic center (24H). Obstet Gynecol*.* (2020) 135:87S–87. 10.1097/01.aog.0000665068.14555.e2

[8] Posnick JC Liu S Tremont TJ. Long-Face dentofacial deformities: occlusion and facial esthetic surgical outcomes. J Oral Maxillofac Surg*.* (2018) 76:1291–1308. 10.1016/j.joms.2017.11.006 29216475

[9] Brauner E Amelina G Laudoni F Fadda MT Armida M De Angelis F. Multidisciplinary planning in orthognathic surgery for prosthetic patients. Appl Sci*.* (2023) 13:10988. 10.3390/app131910988

[10] Elmouden L Ousehal L. Assessment of the quality of life in Moroccan patients undergoing orthognathic surgery. Turk J Orthod*.* (2018) 31:79–85. 10.5152/turkjorthod.2018.17040 30206566 PMC6124887

[11] Psomiadis S Gkantidis N Sifakakis I Iatrou I. Perceived effects of orthognathic surgery versus orthodontic camouflage treatment of convex facial profile patients. J Clin Med*.* (2023) 13:91. 10.3390/jcm13010091 38202096 PMC10780077

[12] Sakhariya SV Waknis PP Setiya S Tidke SS. Effect of induced hypotensive anesthesia and normotensive anesthesia on intraoperative blood loss during orthognathic surgery: a systematic review. J Maxillofac Oral Surg*.* (2023) 23:1127–1137. 10.1007/s12663-023-02034-y 39376768 PMC11456043

[13] Mobini A Mehra P Chigurupati R. Postoperative pain and opioid analgesic requirements after orthognathic surgery. J Oral Maxillofac Surg*.* (2018) 76:2285–2295. 10.1016/j.joms.2018.05.014 29886112

[14] Pathan H Williams J. Basic opioid pharmacology: an update. Br J Pain. (2012) 6:11–16. 10.1177/2049463712438493 26516461 PMC4590096

[15] Meyer M Strazdins E Guessoum A Westenberg JN Appenzeller-Herzog C Cattaneo MEGV. Relative risks of adverse effects across different opioid agonist treatments—a systematic review and meta-analysis. Addiction*.* (2025) 120:1112–1126. 10.1111/add.70000 39924451

[16] Kehlet H. Multimodal approach to control postoperative pathophysiology and rehabilitation. Br J Anaesth*.* (1997) 78:606–617. 10.1093/bja/78.5.606 9175983

[17] Fearon K Ljungqvist O Von Meyenfeldt M Revhaug A Dejong C Lassen K, Enhanced recovery after surgery: a consensus review of clinical care for patients undergoing colonic resection. Clin Nutr*.* (2005) 24:466–477. 10.1016/j.clnu.2005.02.002 15896435

[18] Brenkman H J.F. Roelen S V.S. Steenhagen E Ruurda J P. van Hillegersberg R. Postoperative complications and weight loss following jejunostomy tube feeding after total gastrectomy for advanced adenocarcinomas. Chin J Cancer Res*.* (2017) 29:333–340. 10.21147/j.issn.1000-9604.2017.04.06 28947865 PMC5592821

[19] Joshi Otero J Detriche O Mommaerts MY. Fast-track orthognathic surgery: an evidence-based review. Ann Maxillofac Surg*.* (2017) 7:166–175. 10.4103/ams.ams_44_17 29264281 PMC5717890

[20] Ferrara JT Tehrany GM Chen Q Sheinbaum J Mora-Marquez J Hernandez Conte A. Evaluation of an enhanced recovery after surgery protocol (ERAS) for same-day discharge and reduction of opioid use following bimaxillary orthognathic surgery. J Oral Maxillofac Surg*.* (2022) 80:38–46. 10.1016/j.joms.2021.07.002 34339616

[21] Kou L Wan W Chen C Zhao D Sun X Gao Z. Can the full-percutaneous endoscopic lumbar discectomy in day surgery mode achieve better outcomes following enhanced recovery after surgery protocol? A retrospective comparative study. Front Surg*.* (2022) 9. 10.3389/fsurg.2022.914986 PMC9407013 36034364

[22] Torres M. Effectiveness of multi-modal analgesia. J Perianesth Nurs*.* (2019) 34:e9. 10.1016/j.jopan.2019.05.028

[23] Moussa N Ogle OE. Acute pain management. Oral Maxillofac Surg Clin North Am*.* (2022) 34:35–47. 10.1016/j.coms.2021.08.014 34750009

[24] Joshi GP. Rational multimodal analgesia for perioperative pain management. Curr Pain Headache Rep*.* (2023) 27:227–237. 10.1007/s11916-023-01137-y 37405552

[25] Sarkar S Baliga M Chakraborty S Tusharbhai DM. Re: postoperative pain and opioid analgesic requirements after orthognathic surgery. J Oral Maxillofac Surg*.* (2019) 77:673–674. 10.1016/j.joms.2018.11.037 30641032

[26] Suzen M Zengin M Ciftci B Uckan S. Does the vitamin C level affect postoperative analgesia in patients who undergo orthognathic surgery?. Int J Oral Maxillofac Surg*.* (2023) 52:205–210. 10.1016/j.ijom.2022.06.005 35791994

[27] Joachim M Miloro M. Multimodal approaches to postoperative pain management in orthognathic surgery: a comprehensive review. Int J Oral Maxillofac Surg*.* (2025) 54:914–923. 10.1016/j.ijom.2025.02.004 39966055

[28] Bär AK Werkmeister R Dort JC Al-Nawas B. Perioperative care in orthognathic surgery - A systematic review and meta-analysis for enhanced recovery after surgery. J CranioMaxillofac Surg*.* (2024) 52:1244–1258. 10.1016/j.jcms.2024.08.014 39183122

[29] Xuan C Yan W Wang D Li C Ma H Mueller A. Efficacy of preemptive analgesia treatments for the management of postoperative pain: a network meta-analysis. Br J Anaesth*.* (2022) 129:946–958. 10.1016/j.bja.2022.08.038 36404458

[30] Lavand'homme P. From preemptive to preventive analgesia. Regional anesthesia and pain medicine*.* (2011) 36:4–6. 10.1097/aap.0b013e31820305b8 21455081

[31] Szedlák B Mitre C Fülesdi B. Preemptív és preventív analgesia – a perioperatív fájdalomcsillapítás fontos eleme. Orv Hetil*.* (2018) 159:655–660. 10.1556/650.2018.31045 29681177

[32] Lages LPDD Bergamaschi CDC Lopes LC da Frota EG Silva MT Monte TL. Preemptive oral analgesia with steroidal and nonsteroidal anti-inflammatory drugs in periodontal surgery: a systematic review. Front Pharmacol*.* (2024) 15. 10.3389/fphar.2024.1385401 PMC11285104 39076590

[33] Ramasubbu S Wahab A. Efficacy of preemptive analgesia with pregabalin in orthognathic surgery-A systematic review. J Pharm Res Int*.* (2021) 33:334–340. 10.9734/jpri/2021/v33i43b32560

[34] Canpolat DG Kaba YN Yaşlı SO Demirbaş AE. Using intravenous ibuprofen for preventive analgesia in orthognathic surgery. J Oral Maxillofac Surg*.* (2021) 79:551–558. 10.1016/j.joms.2020.10.029 33197414

[35] Alyahya A Aldubayan A Swennen GR Al-Moraissi E. Effectiveness of different protocols to reduce postoperative pain following orthognathic surgery: a systematic review and meta-analysis. Br J Oral Maxillofac Surg*.* (2022) 60:e1–e10. 10.1016/j.bjoms.2022.03.013 35690502

[36] Oono Y Takagi S Arendt-Nielsen L Kohase H. Perioperative nerve blockade reduces acute postoperative pain after orthognathic surgery. Pain Res Manag*.* (2023) 2023:1–9. 10.1155/2023/7306133 PMC10751169 38149075

[37] Wu Y Liu B Xun Z Yang Y Shang H Zhang H. Do regional nerve blocks during bimaxillary surgery decrease postoperative pain and vomiting compared with patient-controlled analgesia? J Oral Maxillofac Surg*.* (2024) 82:1349–1358. 10.1016/j.joms.2024.07.011 39103152

[38] Esquerré T Mure M Minville V Prevost A Lauwers F Ferré F. Bilateral ultrasound-guided maxillary and mandibular combined nerves block reduces morphine consumption after double-jaw orthognathic surgery: a randomized controlled trial. Reg Anesth Pain Med*.* (2024) 50:575–580. 10.1136/rapm-2024-105497 PMC12322447 38697776

[39] Chen Y Rivera-Serrano C Chen C Chen Y. Pre-surgical regional blocks in orthognathic surgery: prospective study evaluating their influence on the intraoperative use of anaesthetics and blood pressure control. Int J Oral Maxillofac Surg*.* (2016) 45:783–786. 10.1016/j.ijom.2015.09.014 26811189

[40] Baghaie H Shukla K Stone J Breik O Munn Z. Effectiveness of prophylactic tranexamic acid versus placebo or no intervention for reducing blood loss in healthy patients undergoing orthognathic surgery: a systematic review protocol. JBI Evidence Synth*.* (2022) 21:430–440. 10.11124/jbies-22-00126 36081370

[41] Goncin U Liu KK Rawlyk B Dalkilic S Walker MEJ Norton J. Comparison of the ClearSight™ finger cuff monitor versus invasive arterial blood pressure measurement in elective cardiac surgery patients: a prospective observational study. Can J Anesth/J Can Anesth*.* (2024) 71:1495–1504. 10.1007/s12630-024-02834-x 39317830

[42] Haberer J. À propos des revues scientifiques et des bases de données bibliographiques dont disposent les médecins d’un service d’anesthésie–réanimation de centre hospitalier et universitaire en France métropolitaine. Ann Fr Anesth Reanim*.* (2002) 21:182–183. 10.1016/s0750-7658(02)00620-2 11963380

[43] Tewari A Singh G Mishra M Gaur A Mallan D. Comparative evaluation of hypotensive and normotensive anesthesia on LeFort I osteotomies: a randomized, double-blind, prospective clinical study. J Maxillofac Oral Surg*.* (2020) 19:240–245. 10.1007/s12663-019-01325-7 32346234 PMC7176776

[44] Pires B Canguçú D Mendes G Braga A Costa J. Controlled hypotension in orthognathic surgery: a systematic review. J Stomatol Oral Maxillofac Surg*.* (2022) 123:e753–e759. 10.1016/j.jormas.2022.04.013

[45] Barak M Yoav L Abu el-Naaj I. Hypotensive anesthesia versus normotensive anesthesia during Major maxillofacial surgery: a review of the literature. Sci World J. (2015) 2015. 10.1155/2015/480728 PMC4355120 25811042

[46] Shin S Lee JW Kim SH Jung YS OH YJ. Heart rate variability dynamics during controlled hypotension with nicardipine, remifentanil and dexmedetomidine. Acta Anaesthesiol Scand*.* (2013) 58:168–176. 10.1111/aas.12233 24261345

[47] Choi SH Lee WK Lee KY Shin BH Lee SJ. Efficacy of remifentanil-induced controlled hypotension for orthognathic two jaw surgery. Korean J Anesthesiol*.* (2007) 52:62. 10.4097/kjae.2007.52.1.62

[48] Dammling CW Weber TM Taylor KJ Kinard BE. Does tranexamic acid reduce the need for hypotensive anesthesia within orthognathic surgery? A retrospective study. J Maxillofac Oral Surg*.* (2024) 23:229–234. 10.1007/s12663-024-02119-2 38601251 PMC11001797

[49] Baghaie H Shukla K Stone J Breik O Munn Z. Prophylactic tranexamic acid use in orthognathic surgery: a systematic review and meta-analysis. Aesth Plast Surg*.* (2025) 49:2932–2946. 10.1007/s00266-025-04738-7 PMC12222359 40064645

[50] Singh V Bhatt V Dahiya A Bhagol A Sharma A. Role of tumescent solution in modification of the orthognathic surgery: a pilot study. J Maxillofac Oral Surg*.* (2025) 24:725–729. 10.1007/s12663-025-02471-x 40453620 PMC12122398

[51] Yasli S Dogruel F Demirbas A Canpolat D. A case of diffuse alveolar hemorrhage after orthognathic surgery. Niger J Clin Pract*.* (2022) 25:967–970. 10.4103/njcp.njcp_1869_21 35708442

[52] Poon YY Hung KC Chou WY Wang CH Hung CT Chin JC. Is prevention of postoperative vomiting surgery dependent? A retrospective cohort study of total knee arthroplasty. J Pers Med*.* (2021) 11:1018. 10.3390/jpm11101018 34683159 PMC8540625

[53] Grillo R Borba AM Brozoski MA da Silva YS Samieirad S Naclério-Homem MDG. Postoperative nausea and vomiting in orthognathic surgery: systematic review and meta-analysis. Oral Maxillofac Surg*.* (2024) 28:1019–1028. 10.1007/s10006-024-01235-0 38509315

[54] Pourtaheri N Peck CJ Maniskas S Park KE Allam O Chandler L. A comprehensive single-center analysis of postoperative nausea and vomiting following orthognathic surgery. J Craniofac Surg*.* (2021) 33:584–587. 10.1097/scs.0000000000008052 34510064

[55] Dinh KH McAuliffe PF Boisen M Esper SA Subramaniam K Steiman JG. Post-operative nausea and analgesia following total mastectomy is improved after implementation of an enhanced recovery protocol. Ann Surg Oncol*.* (2020) 27:4828–4834. 10.1245/s10434-020-08880-1 32748151

[56] Som A Bhattacharjee S Maitra S Arora MK Baidya DK. Combination of 5-HT3 antagonist and dexamethasone is superior to 5-HT3 antagonist alone for PONV prophylaxis after laparoscopic surgeries. Anesth Analg*.* (2016) 123:1418–1426. 10.1213/ane.0000000000001617 27870735

[57] Aijima R Miura D Takamori A Kamohara A Danjo A Sakaguchi Y. Impact of general anesthesia on postoperative complications in orthognathic surgery: a retrospective comparison of total intravenous anesthesia versus volatile anesthesia. Sci Rep*.* (2024) 14. 10.1038/s41598-024-66926-w 38992157 PMC11239665

[58] Mohamed El-Taher WAM Mohamed MM Ibrahim El Mowafy AY. Midazolam versus ondansetron versus dexamethasone in preventing post-operative nausea and vomiting after elective laparoscopic abdominal surgeries. QJM*.* (2024) 117. 10.1093/qjmed/hcae070.014

[59] Labafchi A Shooshtari Z Grillo R Sharifian Attar v Eshghpour M Samieirad S. The beneficial effect of preoperative dexmedetomidine in controlling postoperative pain, nausea, and vomiting after orthognathic surgery: a triple-blind randomized clinical trial. J Oral Maxillofac Surg*.* (2023) 81:941–949. 10.1016/j.joms.2023.04.014 37209710

[60] Wang LK Cheng T Yang XD Xiong GL Li N Wang DX. Penehyclidine for prevention of postoperative nausea and vomiting following bimaxillary orthognathic surgery: a randomized, double-blind, controlled trial. J Anesth*.* (2021) 36:122–136. 10.1007/s00540-021-03017-4 34738161 PMC8807454

[61] Stratton M Waite P Powell K Scopel M Kukreja P. Benefits of the enhanced recovery after surgery pathway for orthognathic surgery. Int J Oral Maxillofac Surg*.* (2022) 51:214–218. 10.1016/j.ijom.2021.04.008 33966966

[62] Alvarez GA Hebert KJ Britt MC Resnick CM Padwa BL Green MA. An enhanced recovery after surgery (ERAS) protocol for orthognathic surgery reduces rates of postoperative nausea. J Craniofac Surg*.* (2024) 35:1125–1128. 10.1097/scs.0000000000010121 38656374

[63] Gao PF Zhao L Li SY Li Y Chen MK Fu J. Prevention of postoperative nausea and vomiting after orthognathic surgery: a scoping review. BMC Anesthesiol*.* (2024) 24. 10.1186/s12871-024-02510-z PMC10976820 38539078

[64] Yang D Grant MC Stone A Wu CL Wick EC. A meta-analysis of intraoperative ventilation strategies to prevent pulmonary complications. Ann Surg*.* (2016) 263:881–887. 10.1097/sla.0000000000001443 26720429

[65] Borges JB Amato MBP Hedenstierna G. The increasing call for protective ventilation during anesthesia. JAMA Surg*.* (2017) 152:893. 10.1001/jamasurg.2017.1614 28593243

[66] Coppola S Froio S Chiumello D. Protective lung ventilation during general anesthesia: is there any evidence?. Crit Care*.* (2014) 18. 10.1186/cc13777 PMC4057231 25029254

[67] Ferrando C Vallverdú J Zattera L Tusman G Suárez-Sipmann F. Improving lung protective mechanical ventilation: the individualised intraoperative open-lung approach. Br J Anaesth*.* (2025) 134:281–287. 10.1016/j.bja.2024.10.007 39880492

[68] Müller-Wirtz LM O’Gara B Gama de Abreu M Schultz MJ Beitler JR Jerath A. Volatile anesthetics for lung- and diaphragm-protective sedation. Crit Care*.* (2024) 28. 10.1186/s13054-024-05049-0 PMC11366159 39217380

[69] Fernandes M Eufrásio A Bonifácio J Marcelino J. Airway management in crouzon syndrome: a challenge for the anaesthetist. BMJ Case Rep*.* (2018) 2018:bcr-2017-219371. 10.1136/bcr-2017-219371 PMC5976076 29848516

[70] Hart PS McIntyre BP Kadioglu O Currier GF Sullivan SM Li J. Postsurgical volumetric airway changes in 2-jaw orthognathic surgery patients. Am J Orthod Dentofacial Orthop*.* (2015) 147:536–546. 10.1016/j.ajodo.2014.12.023 25919099

[71] Salerno A Hermann R. Efficacy and safety of steroid use for postoperative pain relief. J Bone Joint Surg*.* (2006) 88:1361–1372. 10.2106/jbjs.d.03018 16757774

[72] Kormi E Snäll J Törnwall J Thorén H. A survey of the use of perioperative glucocorticoids in oral and maxillofacial surgery. J Oral Maxillofac Surg*.* (2016) 74:1548–1551. 10.1016/j.joms.2016.02.027 27019411

[73] Abukawa H Ogawa T Kono M Koizumi T Kawase-Koga Y Chikazu D. Intravenous dexamethasone administration before orthognathic surgery reduces the postoperative edema of the masseter muscle: a randomized controlled trial. J Oral Maxillofac Surg*.* (2017) 75:1257–1262. 10.1016/j.joms.2016.12.048 28157491

[74] Lin HH Kim SG Kim HY Niu LS Lo LJ. Higher dose of dexamethasone does not further reduce facial swelling after orthognathic surgery. Ann Plast Surg*.* (2017) 78:S61–S69. 10.1097/sap.0000000000001008 28118231

[75] Bravo M Bendersky Kohan J Uribe Monasterio M. Effectiveness of glucocorticoids in orthognathic surgery: an overview of systematic reviews. Br J Oral Maxillofac Surg*.* (2022) 60:e231–e245. 10.1016/j.bjoms.2021.04.011 35067412

[76] Gupta K Waknis PP. Postoperative steroid dosing in orthognathic surgery: a narrative review of literature. J Oral Maxillofac Surg Med Pathol*.* (2023) 35:305–307. 10.1016/j.ajoms.2022.12.008

[77] Corcoran TB Myles PS Forbes AB O'Loughlin E Leslie K Story D. The perioperative administration of dexamethasone and infection (PADDI) trial protocol: rationale and design of a pragmatic multicentre non-inferiority study. BMJ Open*.* (2019) 9:e030402. 10.1136/bmjopen-2019-030402 31494615 PMC6731833

[78] Wolff CB Green DW. Clarification of the circulatory patho-physiology of anaesthesia – implications for high-risk surgical patients. Int J Surg*.* (2014) 12:1348–1356. 10.1016/j.ijsu.2014.10.034 25448657

[79] Shah KSV Fernando S Preena D Jigajinni S Ahmed A. 1318 Peri And post-operative fluid management in relation to outcomes in free flap reconstructive surgery - A systematic review. Br J Surg*.* (2021) 108. 10.1093/bjs/znab259.633

[80] González J Andrés G Martínez-Salamanca JI Ciancio G. Improving surgical outcomes in renal cell carcinoma involving the inferior vena cava. Expert Rev Anticancer Ther*.* (2013) 13:1373–1387. 10.1586/14737140.2013.858603 24236819

[81] Heming N Moine P Coscas R Annane D. Perioperative fluid management for major elective surgery. Br J Surg*.* (2020) 107:e56–e62. 10.1002/bjs.11457 31903587

[82] Vallurupalli M Fligor J Shah ND Pham L Pfaff MJ Vyas RM. Assessing use and familiarity of enhanced recovery after surgery elements in pediatric orthognathic surgery. J Craniofacial Surg*.* (2024) 36:224–228. 10.1097/scs.0000000000010749 39724594

